# Noninvasive Monitoring of Simulated Hemorrhage and Whole Blood Resuscitation

**DOI:** 10.3390/bios12121168

**Published:** 2022-12-14

**Authors:** Jay F. Gupta, Saaid H. Arshad, Brian A. Telfer, Eric J. Snider, Victor A. Convertino

**Affiliations:** 1 Lincoln Laboratory, Massachusetts Institute of Technology, Lexington, MA 02421, USA; 2 U.S. Army Institute of Surgical Research, San Antonio, TX 78234, USA

**Keywords:** signal processing, machine learning, hemorrhage, resuscitation, prehospital, prolonged field care, arterial blood pressure waveform, photoplethysmogram

## Abstract

Hemorrhage is the leading cause of preventable death from trauma. Accurate monitoring of hemorrhage and resuscitation can significantly reduce mortality and morbidity but remains a challenge due to the low sensitivity of traditional vital signs in detecting blood loss and possible hemorrhagic shock. Vital signs are not reliable early indicators because of physiological mechanisms that compensate for blood loss and thus do not provide an accurate assessment of volume status. As an alternative, machine learning (ML) algorithms that operate on an arterial blood pressure (ABP) waveform have been shown to provide an effective early indicator. However, these ML approaches lack physiological interpretability. In this paper, we evaluate and compare the performance of ML models trained on nine ABP-derived features that provide physiological insight, using a database of 13 human subjects from a lower-body negative pressure (LBNP) model of progressive central hypovolemia and subsequent progressive restoration to normovolemia (i.e., simulated hemorrhage and whole blood resuscitation). Data were acquired at multiple repressurization rates for each subject to simulate varying resuscitation rates, resulting in 52 total LBNP collections. This work is the first to use a single ABP-based algorithm to monitor both simulated hemorrhage *and* resuscitation. A gradient-boosted regression tree model trained on only the half-rise to dicrotic notch (HRDN) feature achieved a root-mean-square error (RMSE) of 13%, an R^2^ of 0.82, and area under the receiver operating characteristic curve of 0.97 for detecting decompensation. This single-feature model’s performance compares favorably to previously reported results from more-complex black box machine learning models. This model further provides physiological insight because HRDN represents an approximate measure of the delay between the ABP ejected and reflected wave and therefore is an indication of cardiac and peripheral vascular mechanisms that contribute to the compensatory response to blood loss and replacement.

## 1. Introduction

Hemorrhage is the leading cause of preventable death among combat and civilian trauma casualties [1,2,3,4]. Monitoring blood loss to predict hemorrhagic shock, together with intravenous fluid resuscitation, is fundamental to reducing mortality and morbidity in the crucial moments after injury [5]. In the austere setting of tactical combat casualty care, the standard for assessing hemorrhagic shock and circulation volume status is based on “altered mental status in the absence of brain injury and/or weak or absent radial pulse.” [6]. When vital signs are available, these are used to monitor for shock, with the shock index as a longstanding example [7]. Other more sophisticated methods based on vital signs have been developed [8,9,10]. However, the body compensates to maintain vital signs such as heart rate and blood pressure until near the point of shock (~15–20% or more blood volume loss [11,12]), so these measures are not highly sensitive for early prediction of decompensation or recovery from shock [10,13]. Furthermore, inability to accurately monitor hemodynamic status makes it difficult to provide goal-directed resuscitation. Poorly defined resuscitation endpoints can increase morbidity and mortality because of impacts on coagulation, toxicity, and reperfusion injuries [3,13]. Over-resuscitation can further stress a patient’s organs and result in outcomes potentially even more detrimental than that of under-resuscitation [14]. The ability to noninvasively track circulating volume status and accurately differentiate between states of near-shock versus normovolemia is thus crucial for providing effective pre-hospital trauma care.

A variety of alternative measures relying on noninvasive physiological signals that represent compensatory responses that change during the early stages of compensated shock during hemorrhage and the early stages of resuscitation have been explored. Analysis of arterial blood pressure (ABP) waveforms [10,15,16,17,18], detection of dynamic light scattering [19], and seismography [7,15] have been some promising methods to track hemorrhage. The compensatory reserve [14], based on a machine learning (ML) algorithm that interrogates ABP waveform features, has been particularly promising and has been used to track both hemorrhage and resuscitation [3,13,15]. Compensatory reserve is an assessment of the body’s ability to respond to acute changes in circulating blood volume by activating compensatory mechanisms such as tachycardia and vasoconstriction [15]. The ABP waveform is particularly informative because it is affected by both reduced cardiac output and compensatory mechanisms during blood loss.

Compensatory reserve is a physiological phenomenon that represents the fraction of the compensating capacity of an organism in response to blood loss that remains before reaching decompensation (i.e., failure of physiological compensatory mechanisms) [20]. Two specific algorithms have been evaluated in multiple studies for the purpose of monitoring blood loss and predicting hemorrhagic shock. The Compensatory Reserve Index (CRI) uses a proprietary machine learning method [21] and estimates compensatory reserve on a scale of 1 to 0, with 1 corresponding to normovolemia and 0 to a state of decompensation. The second algorithm, termed Compensatory Reserve Metric (CRM), uses a convolutional neural network (CNN) [22] and is reported on a scale of 100% to 0% for normovolemia and decompensation, respectively.

The ability to objectively monitor fluid resuscitation has received less attention than has monitoring blood loss. Convertino et al. reported high correlations between CRM and percent blood lost and resuscitated in nonhuman primates. They calculated a clinically meaningful threshold of systemic oxygen delivery (DO_2_) of 5.3 mL O_2_·kg^−1^·min^−1^ and accordingly proposed an endpoint for resuscitation of CRM ~40% [3,23]. Li et al. demonstrated the ability to track resuscitation using a ML model with feature sets extracted from electrocardiogram (ECG) and photoplethysmogram (PPG) waveforms using a porcine model of induced hemorrhagic shock and fluid resuscitation [14]. They evaluated an algorithm that classified whether a sufficient point of resuscitation had been reached or not and achieved AUCs of 0.892 and 0.947 without and with a personal baseline normalization, respectively.

While these results have been quite promising, black box machine learning approaches do not readily explain the details of the information that is being used and what information is most important. In particular, the physiology of compensation and decompensation is well understood [24], but insight into how CRI and CRM algorithms utilize the information embedded in the ABP waveform remains limited. Interpretability of ML models is advantageous for galvanizing trust and accelerating adoption in high-stakes domains, such as trauma care. This is particularly true when factors utilized by the models are congruous with domain insights held by expert end-users [25].

Previous work has demonstrated the high sensitivity and specificity of a particular ABP-derived feature, the half-rise to dicrotic notch (HRDN), in tracking simulated hemorrhage in a stepped-LBNP model of progressive central hypovolemia [26]. HRDN measures the approximate delay between the ejected and reflected ABP wave components and is known from other research to correlate with the compensatory measure of reduced arterial compliance and vasoconstriction [27,28,29]. The focus of this paper is to build on this previous work by evaluating the relative importance of ABP-derived features in tracking both hemorrhage and resuscitation, thereby elucidating further insights about the information contained within ABP waveforms. The resulting models are alternate approaches for estimating CRM, compared to the method for computing CRM reported in [21]. Three key contributions of this paper are that these physiologically interpretable models: (1) yield similar accuracies for monitoring simulated blood loss compared to more complex, black-box models, (2) yield similar accuracies for both blood loss and fluid resuscitation, and (3) perform similarly for data from both the stepped-LBNP protocol that has been evaluated in past publications and for a new ramped-LBNP protocol that simulates both hemorrhage and resuscitation at different rates.

## 2. Materials and Methods

### 2.1. Hemorrhage Model

An LBNP model was used to simulate whole blood hemorrhage physiology and subsequent resuscitation in healthy human volunteers. Subjects were positioned supine inside a pressure chamber enclosing the lower extremities, with the upper body above the waist exposed to ambient conditions. Progressively lowering the chamber pressure to sub-atmospheric levels reduced central blood volume by redistributing blood toward the lower extremities. LBNP provided a noninvasive method for inducing hemodynamic decompensation and hemorrhagic shock-like symptoms. Returning the chamber to atmospheric pressure at a fixed rate steadily reversed these effects and restored subjects to their baseline physiological state [24].

LBNP studies were conducted at the U.S. Army Institute of Surgical Research under approval from the U.S. Army Medical Research and Development Command Institutional Review Board. LBNP chamber pressures and ABP recordings from 13 subjects were de-identified and provided to MIT Lincoln Laboratory under a data sharing agreement for analysis. Demographic information is summarized in Table 1.

**Figure 1:**
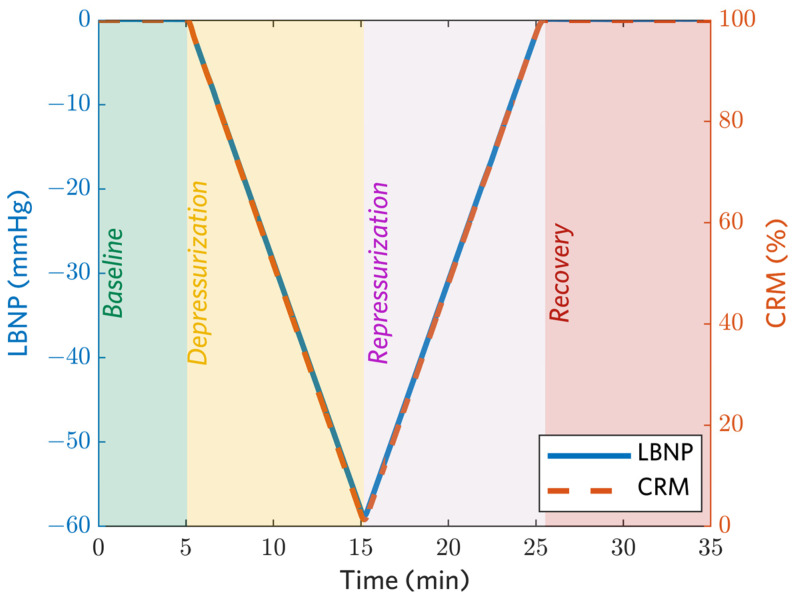
Representative negative pressure application protocol and reference CRM (RCRM) values during the baseline, depressurization, repressurization, and recovery phases of the LBNP studies. The duration of each phase varied based on the ramp speed selected for that trial.

Data were collected from each subject on two separate days. On the first day, negative chamber pressure was applied (i.e., the chamber was depressurized) in a linear ramp at a randomly assigned rate of −3, −6, or −9 mmHg/min to simulate a relatively slow, medium, or fast rate of bleeding. This was consistent with previous experiments demonstrating that −30, −60, and −90 mmHg LBNP approximate average blood losses of 450, 1000, and 1600 mL, respectively in a 70 kg human [22]. Pressure was quickly released (within 2 s) when subjects reached their hemodynamic decompensation point (indicated by a systolic blood pressure (SBP) of 80 mmHg or less, a sudden drop in heart rate (HR), symptoms consistent with clinical criteria of class III shock [15], or sustained an LBNP of −100 mmHg). On the second day, subjects participated in three consecutive LBNP sessions. In each session, chamber pressure was reduced at the same rate assigned to that subject on the first day, until a CRM of 30% was measured or an LBNP of −100 mmHg was reached. The chamber was then gradually repressurized to simulate fluid resuscitation at a rate of either +3, +6, or +9 mmHg/min. The repressurization rate of the first session was the same as the depressurization rate, while the repressurization rates of the second and third sessions were randomly assigned from the remaining options. The repressurization phase was followed by a 10 min recovery period at constant chamber pressure isobaric with ambient conditions. A schematic of the LBNP application protocol is provided in Figure 1. After recovery, subjects were removed from the LBNP chamber for a 60 min rest period between sessions. During the rest period, subjects were provided with water and a light snack and were only allowed to perform non-strenuous or non-stressful activities such as reading a book or watching a movie. No acclimatization was noticed during the multiple sessions. In summary, 13 subjects each participated in 4 sessions of data collection, resulting in a total of 13 depressurization and 39 repressurization sessions.

A beat-to-beat arterial blood pressure signal was noninvasively monitored continuously throughout the LBNP study using photoplethysmography with a Finapres technology (Finometer^®^ Blood Pressure Monitor, BMEye, Amsterdam, The Netherlands) [30]. Data were acquired at a 500 Hz sampling rate. Reference CRM values for each subject were computed as a function of negative pressure level, P, at each time point, t, using Equation (1) as illustrated in Figure 1, where $P_{max}$ is the maximum pressure sustained by the subject:

(1) $$Reference\;CRM_{t}=\left(1-\frac{P_{t}}{P_{max}}\right)*100\%$$

Reference CRM values were used as truth data for training and testing models to estimate CRM.

### 2.2. ABP Signal Processing

#### 2.2.1. Pre-Processing

The Finapres ABP recordings were smoothed by a 512th order zero-phase finite impulse response lowpass filter with 12 Hz cutoff frequency (MATLAB, Natick, MA, USA). First and second derivatives were approximated as finite differences of the denoised ABP traces. Signals and derivatives were z-scored within a 2-s trailing window to detrend, remove baseline drift, and standardize amplitude scaling.

#### 2.2.2. Feature Extraction

**Figure 2:**
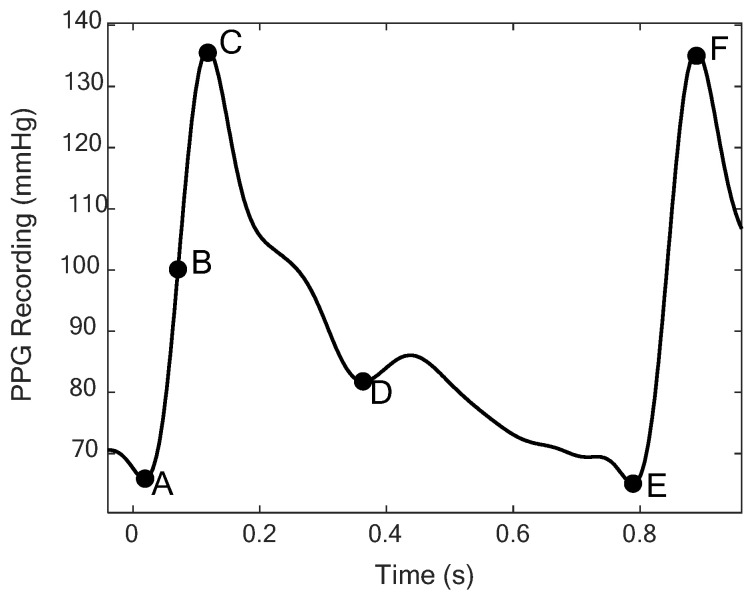
Landmark points on Finapres ABP pulse corresponding to (A) start of pulse, (B) systolic half-rise, (C) systolic peak, (D) dicrotic notch, (E) end of pulse, and (F) systolic peak of successive pulse.

Fiducial points corresponding to the landmarks in Figure 2 were identified based on peak finding (MATLAB 2021b) and empirical relationships between the ABP signal and its derivatives, as described in [27]. Features describing the morphology of each ABP pulse were then computed using the formulas in Table 2 [27]. These features reflect interpretable physiological correlates such as cardiac output, autonomic function, and peripheral vascular resistance [15,26,31]. The feature set also includes standard vital sign features such as heart rate (HR), systolic blood pressure (SBP), and diastolic blood pressure (DBP).

Locating the dicrotic notch is requires additional selection logic when multiple candidate notches are detected, or none are detected. These notches are detected based on the first derivative, as described in [27]. For cases where multiple candidate notches were identified from first-derivative peaks, the maximally prominent peak within a credible time range (15–40% of PPI, based on exploratory analysis) was selected. ABP pulses where no peaks met these criteria were excluded from analysis.

#### 2.2.3. Outlier Rejection

Outlier values for each feature were detected using a moving median filter within a centered, 20-heartbeat window. Data points greater than three scaled median absolute deviations (MAD) away from the window median were removed. Scaled MAD was calculated as 1.4826×median(|A−median(A)|) for each sequence, A, of local feature values [32].

#### 2.2.4. Local Averaging

To reduce the impact of short-term fluctuations, a moving average filter was applied to each feature with a trailing window size of 20 s and a 90% overlap between adjacent windows. Moving standard deviation of the feature averages (centered bilaterally across 21 adjacent windows) was computed for the half-rise to dicrotic notch (HRDN) feature. To remove remaining outliers, samples where the HRDN standard deviation exceeded a threshold value were removed from the data set. This method was empirically tuned to exclude outlier groups from specific subjects identified during exploratory data analysis. Since the computations described above require 20 s of “future” data in the current implementation, a 20 s time delay in reporting results will ensure that the processing pipeline remains causal when the algorithm is deployed in the field.

#### 2.2.5. Baseline Normalization

To evaluate the need for a priori information about an individuals’ normovolemic physiological state, baseline-normalized features were compiled for each subject. To generate these data, each feature was divided by its mean across the five-minute baseline LBNP phase (Figure 1).

### 2.3. CRM Estimation

CRM was estimated as a supervised regression task using the ABP-derived features as model inputs. Ordinary least squares (OLS) and gradient boosted (GB) regression tree [33] models were evaluated in order to interrogate linear and non-linear relationships between the variables, respectively. All tree ensembles consisted of 100 estimators trained to minimize squared error loss without bootstrap aggregation of samples for the base learners. Four different feature sets were evaluated: (1) all nine features listed in Table 2, (2) vital signs (PPI, SBP, HRV, SI, and DBP), (3) ABP-waveform features including several that are not considered as vital signs (PPI, HRDN, PP, and IPA), and (4) HRDN only.

Models were trained on data sets generated with and without baseline normalization. Five-fold cross validation was used to assess model generalizability. Given that samples from the same individual are highly correlated, folds were split by subject in order to avoid data leakage [34]. All regression experiments used the scikit-learn package for machine learning in Python [35]. Models were trained and tested on three versions of the data set:

1. Full procedure: Baseline, depressurization, repressurization, and recovery phases of data collection are included for training and testing.
2. Simulated hemorrhage: Only depressurization data (the phase shaded yellow in Figure 1) are used for training and testing.
3. Simulated resuscitation: Only repressurization data (the phase shaded purple in Figure 1) are used for training and testing.

Training and testing only on the simulated hemorrhage and resuscitation phases allowed evaluation of the models’ ability to accurately track compensatory reserve with comparable performance during both phases of monitoring a subject.

### 2.4. Analysis and Performance Metrics

Gini importance [35] was examined to determine the relative contribution of each feature in the GB regression tree models. Importance was calculated as the normalized decrease in Friedman’s MSE criterion [33] after performing splits with the feature being evaluated.

Bland–Altman (BA) analysis [36] was also conducted to assess agreement between reference and estimated CRMs. Given that gold standard reference values were known from Equation (1), the *x*-axis was selected as the reference CRM [37].

Model accuracy was quantified by RMSE, coefficient of determination (R^2^) between estimated and reference CRM, and ROC AUC. The effect of variable rates of simulated hemorrhage and resuscitation were also evaluated by averaging RMSE and R^2^ per ramp speed: −9, −6, −3, +3, +6, and +9 mmHg/min, where the negative rates indicate the depressurization (simulated hemorrhage) and the positive rates indicate repressurization (simulated resuscitation). For AUC, ROCs were computed for binary classification at the clinically relevant CRM thresholds of 40% and 70% based on the work of Koons et al. [23]. A CRM of 40% corresponds to the critical threshold at which reduction in DO_2_ places individuals with low tolerance to reductions in central blood volume at greatest risk for imminent hemodynamic decompensation. A CRM of 70% corresponds to the recommended safe range that allows return to normovolemia without risk of over-resuscitation. To allow comparison to previously reported AUCs at the point of decompensation, an AUC was also computed for a CRM threshold of 5%, which is slightly higher than the decompensation point of CRM = 0% to allow for low-level noise in the CRM estimates.

## 3. Results

### 3.1. Performance of CRM Estimation Models

Subject 12 had only the initial depressurization data and no repressurization data. Of the total data available, 2.8% was removed after the median outlier rejection step and 4.3% was removed after the HRDN standard deviation threshold step. Table 3 presents performance metrics for a GB tree model trained and tested on four different feature sets for the full procedure, only the depressurization phase, and only the repressurization phase as depicted in Figure 1. A simple linear regression on the HRDN feature was also evaluated, but this performed poorly relative to the GB tree models and is not shown. Without baseline normalization, the highest performing models (indicated by lowest RMSE and highest R^2^) were the GB tree trained on all features using the data from the full procedure for each subject with RMSE = 13% ± 2%, R^2^ = 0.85 ± 0.04, and the GB tree trained on the ABP waveform feature set with RMSE = 13% ± 2%, R^2^ = 0.85 ± 0.04. The vital signs feature set has the lowest performance with an RMSE = 23% ± 0.03%, R^2^ = 0.50 ± 0.14. The model using only the HRDN feature achieved comparable performance to the aforementioned high-performance models, suggesting that the HRDN feature by itself is an accurate estimator of CRM. Similar trends were observed for models trained and tested just on the depressurization (simulated hemorrhage) and repressurization phases; however, overall performance of these models was worse compared to training and testing using data from the full procedure.

It should be noted that the HRDN feature decreases as PPI decreases (i.e., as heart rate increases), so it is natural to consider the normalized feature HRDN/PPI. We evaluated that feature but found no performance improvement.

The models using the vital signs feature set improved considerably after applying baseline normalization, whereas there was no marked improvement for the other feature set configurations. The GB tree models using HRDN showed a decrease in performance after baseline normalization in the case of training on full procedure data or repressurization data. This performance decrease was due to two subjects with significant variability in ABP waveform data during the baseline phase of the LBNP study, which resulted in inaccurate HRDN features in the baseline phase.

**Figure 3:**
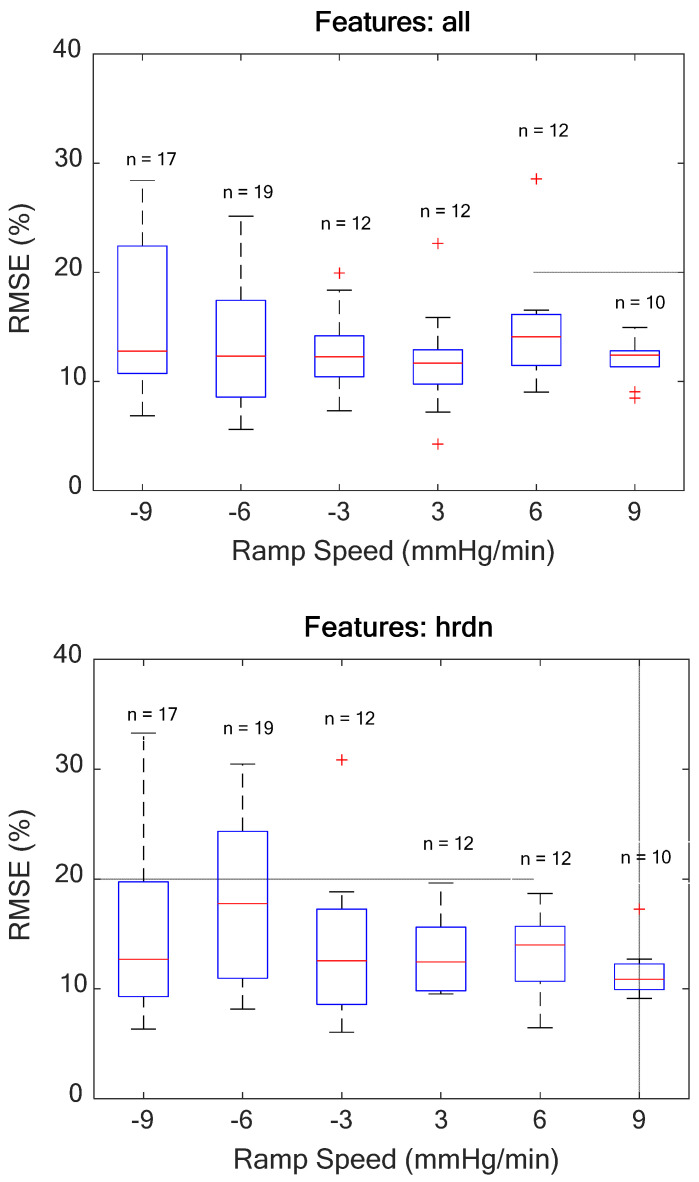
Box plots showing statistical characteristics of the RMSEs binned per ramp speed. The median (red line), 25th and 75th percentiles (top and bottom edges of the blue box), outliers (red crosses), and valid maximum and minimum values that were not classified as outliers (whiskers). The top plot shows the RMSEs for the all-features GB tree model binned without baseline normalization per ramp speed, while the bottom plot shows the same data for the model that only uses HRDN.

Figure 3 shows box plots of RMSEs at each ramp speed for the GB tree model with all features and for the GB tree model using only HRDN. The number of data sets per speed was non-uniform. Ramp rates −9, −6, −3, +3, +6, and +9 mmHg/min had 18, 19, 12, 12, 12, and 11 data sets, respectively. In the case of the all-features set, maximum variability in RMSE across subjects was seen at −9 mmHg/min with a standard deviation of 7%, and minimum variability was observed at 9 mmHg/min with a 2% standard deviation. Median values for all speeds fell within one standard deviation of each other and there were no significant differences in performance between the all-features set and HRDN-only set with regard to speed. This suggests that the reliability of HRDN as a feature for tracking hemorrhage and resuscitation is independent of rate of blood volume and/or fluid resuscitation.

**Figure 4:**
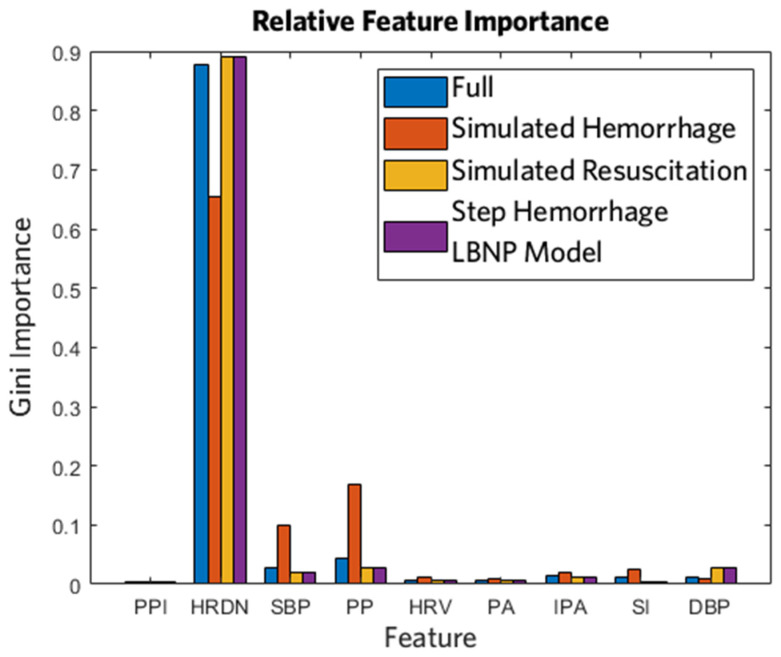
Gini importance plots for full procedure model, simulated hemorrhage model, simulated resuscitation model, and step hemorrhage LBNP protocol model [26]. These plots are for the GB tree model trained using all features without any baseline normalization.

Figure 4 shows the relative importance of all features tested in the GB tree models without baseline normalization for each version of the data set. Figure 4 shows the same analysis for the equivalent model from previous work that utilized a stepped-LBNP protocol to simulate progressive hemorrhage, instead of the ramped-LBNP protocol employed in this paper [26]. HRDN was identified as the primary contributor in all cases, whether it was being used to estimate CRM during simulated hemorrhage or simulated resuscitation.

**Figure 5:**
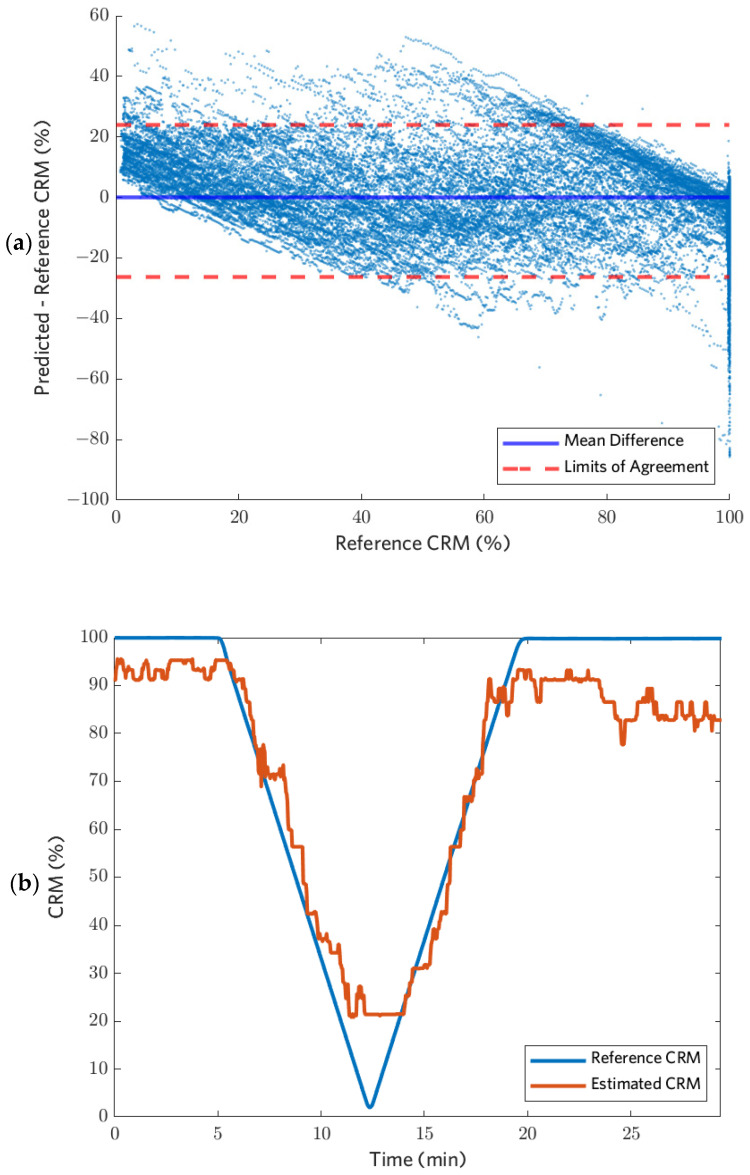
(**a**) A BA plot is shown for the GB tree model trained using all features without baseline normalization for all subjects, and (**b**) Sample reference CRM and estimated CRM using a GB tree model trained with only the HRDN feature from subject 4 without baseline normalization. This example has a lower than average RMSE of 9.9%.

Figure 5 shows a BA plot, as well as reference and estimated CRMs from an example subject, for the GB tree model trained on the full procedure data set using all nine features in Table 2. The limits of agreement (representing ±1.96 standard deviations) were at ~25% difference between the estimated and reference CRM for all models tested, except for the vital signs models, for which they were ~40%. Overall, the model bias was negatively correlated with CRM. It tended to undershoot for higher CRMs (>50%) and overshoot for lower CRMs (<50%), despite low mean error across the full dataset. Figure 5b shows the CRM estimates for subject 4 during a session with the simulated hemorrhage and resuscitation induced at the same speed (~6 mmHg/min). This trend was observed in all iterations of models tested and is expected for a least-squares-fit model.

### 3.2. ROC Analysis for Classification at Key Clinical Endpoints

Table 4 presents results for the ROC analysis and AUCs for all model configurations trained and tested as shown in Table 3. AUCs were calculated for binary classification at empirically determined CRM thresholds (i.e., CRM ≥ 70%, CRM ≥ 40%, and CRM ≥ 5%) that have been proposed as clinical endpoints for management of both hemorrhage and resuscitation. Overall, every model utilizing all features listed in Table 2 performed well in each of these binary classification tasks.

ROC AUCs trended with the RMSE and R^2^ metrics in Table 3, so models showing a lower RMSE and higher R^2^ in Table 3 correspondingly showed to a relatively higher AUC in Table 4. The GB tree model trained on the vital signs feature set without baseline normalization had the lowest AUC of 0.78 ± 0.06. Of the 72 models trained, 57 achieved an AUC above 0.9, however lower AUCs were observed for detecting CRM ≥ 5% compared to the other two endpoints. The HRDN-only models performed similarly to the models trained on all features, showing that this single ABP-derived feature is sufficient to distinguish between key CRM thresholds with high sensitivity and specificity during both hemorrhage and resuscitation.

## 4. Discussion

Although LBNP ramp protocols have been used for investigation of the hemodynamic responses to progressive central hypovolemia [38], this is the first study in which the compensatory reserve has been analyzed with a constant ramp LBNP protocol that simulates both hemorrhage and resuscitation. Most previous LBNP protocols were designed to depressurize the vacuum chamber using a step profile in which negative pressure was maintained at a certain value [15,16,23] for a fixed period of time between depressurization steps. The ramped protocol applies a constant rate for both depressurization and repressurization, more accurately simulating both scenarios of whole blood hemorrhage or resuscitation, which are continuous processes in reality. Furthermore, the analysis presented in the present article is based on the same algorithm for both hemorrhage and resuscitation. This indicates that the underlying physiological mechanisms that are being measured by a feature such as HRDN remain valid in measuring compensatory reserve in both scenarios. This relationship can therefore rely on employing only a single algorithm in the field when managing hemorrhage.

This present investigation is also the first work to assess the effect of the *rate* of simulated hemorrhage and resuscitation on the accuracy of a model using an LBNP protocol. As Figure 3 shows, there are no statistically significant differences in RMSE across the different rates of hemorrhage or resuscitation. The HRDN-only model shows a similar accuracy and robustness to speed as the all-features model, further demonstrating the efficacy of the HRDN-only model. A limitation in this study is the non-uniform distribution of rates and a relatively small population size, which are factors that will be addressed as the data set grows.

Our work builds on recent preliminary results for resuscitation monitoring, particularly in relating metrics such as CRI and CRM to real physiological conditions [13,15,28,39,40]. Koons et al. demonstrated in a study on baboons that CRM is linearly correlated to DO_2_ during controlled progressive hemorrhage and subsequent whole blood resuscitation [3]. Using this relationship, they showed that hemodynamic decompensation (CRM = 0%) can be defined by a critical DO_2_ at approximately 5.3 mL O_2_∙kg^−1^∙min^−1^. Furthermore, using this model they showed that a target CRM of 35% during whole blood resuscitation required only 40% of total blood volume lost to sustain adequate DO_2_ to maintain hypotensive resuscitation [3]. Their most recent work establishes a CRM of 40% as a key threshold to ensure a patient at greatest risk for early onset of shock (i.e., individual with low tolerance to central hypovolemia) is at an adequate DO_2_ level [23]. Unique to the current study is the varying rates of simulated whole blood hemorrhage and resuscitation used to develop the HRDN model. Given HRDN’s high performance in predicting CRM in the LBNP model, there is potential for HRDN to be correlated with a clinically meaningful metric such as DO_2_. Given the HRDN-only model’s accurate tracking of CRM and high discriminability at the key thresholds established by Koons et al., the models explored in this paper could be implemented in ambulatory and austere field-care environments working off noninvasive PPG data. The HRDN-only models furthermore provide a clinically interpretable result, relating the time difference between the ejected and reflected waves in a PPG signal and providing information on systemic vascular resistance, which will change dramatically in response to hemorrhage and resuscitation [26].

Recent work by Convertino et al. [22] demonstrated accurate tracking of reductions in central blood volume similar to those produced by hemorrhage induced by a stepped-LBNP model with a cohort of 191 healthy human volunteers using CRM and CRI. The algorithms estimated the onset of hemodynamic decompensation with a CRI AUC of 0.9164 (0.0066, 95% CI = 0.903–0.92) with an R^2^ = 0.978 and a CRM AUC of 0.9268 (0.0059, 95% CI = 0.915–0.93) with an R^2^ = 0.958. The investigators did not simulate resuscitation in this study. The HRDN-only GB tree models reported herein achieved AUCs of 0.93 ± 0.02, 0.82 ± 0.08, 0.88 ± 0.03, and corresponding R^2^s of 0.82 ± 0.07, 0.67 ± 0.12, and 0.80 ± 0.09 on the full procedure, only depressurization, and only repressurization versions of the data set, respectively. The performance of our simplified single feature model is thus comparable with respect to AUC. The R^2^ values here are lower, with the maximum reaching only 0.82 compared to 0.978 for CRI and 0.958 for CRM. We expect that this would improve with a larger data set. While the data set analyzed in this paper was limited to 13 subjects with 52 LBNP collections compared to 191 subjects in the study by Convertino et al. [22], our work underscores the ability to track both hemorrhage and resuscitation and demonstrates that the models presented in this current investigation track both with comparable accuracy, even when using only HRDN as a feature.

This work supports the sound physiological basis for the concept of a compensatory reserve metric. Previous work by Convertino et al. has established the efficacy of estimating compensatory reserve in a variety of human LBNP models using a step protocol [15,16,20,21,23,24,38]. This model has been shown to be an accurate simulation of real blood loss and resuscitation in humans and primates [3,10,16,29,39]. We have demonstrated an HRDN-based model with comparable performance on both an LBNP step protocol [26] and the ramp protocol presented in this paper. The similar and accurate performance of the CRI, CRM, and HRDN models on step and ramp protocols for both simulated hemorrhage and resuscitation provides compelling evidence for the measurement of compensatory reserve as a valid physiological concept for hemorrhage management.

## 5. Conclusions

The present study has demonstrated analysis of a novel data set simulating both whole blood hemorrhage and resuscitation at varying rates. The HRDN feature previously reported [26] and used in a GB tree model tracks hemorrhage and resuscitation with similar accuracies and comparable performance to that from more complex deep learning-based systems reported elsewhere. The HRDN feature is physiologically interpretable, relating the time between the ejected and reflected pressure waves to systemic vascular resistance and cardiac output. The HRDN model accurately classifies key clinical thresholds for managing hemorrhage and resuscitation, and importantly does not require baseline normalization that would be difficult to obtain in typical clinical settings. Finally, it has shown to maintain performance in hemorrhage and resuscitation rates varying from 3 to 9 mmHg/min in an LBNP model. The work presented here demonstrates a promising solution for monitoring hemorrhage and resuscitation in ambulatory and austere field-care settings. The key limitations of this work are a small subject sample size and a non-uniform distribution of rates of simulated hemorrhage and resuscitation. Validation of the HRDN model generated by the analysis of varying ramp protocols in the present investigation awaits future work that focuses on applying our HRDN models to actual blood loss and correlating values of compensatory reserve with clinically meaningful metrics such as DO_2_.

## Figures and Tables

**Table 1:** Study Cohort Demographics.

Factor	Subject Group ^a^
	All Subjects
Gender	6 male, 7 female
Age	23 ± 4 years
Weight	68.5 ± 11.6 kg
Height	173 ± 9 cm
Body Mass Index	23.1 ± 3.9

^a^. Demographics presented as mean ± standard deviation, where applicable.

**Table 2:** ABP Feature Descriptions.

Feature	Description	
	Name	Formula ^a^
PPI	Peak to Peak Interval	$t_{F}-t_{C}$
HRV	Heart Rate Variability	RMSSD of PPI for 10 beats
HRDN	Half-Rise to Dicrotic Notch	$t_{D}-t_{B}$
SBP	Systolic Blood Pressure	$W_{C}$
DBP	Diastolic Blood Pressure	$W_{E}$
PP	Pulse Pressure	$W_{C}-W_{A}$
PA	Pulse Area	$\int_{A}^{E}W\;dt$
IPA	Inflection Point Area	$\int_{D}^{E}W\;dt\;÷\;\int_{A}^{D}W\;dt$
SI	Shock Index	$\frac{60}{PPI\cdot SBP}$

^a^. $W_{x}$ is the ABP signal value at point x and $t_{x}$ is the time at point x, based on the schematic in Figure 2.

**Table 3:** CRM estimation results for cross-validation training and testing for the GB-tree model on the full data set (includes the baseline, depressurization, repressurization, and recovery phases from each experiment as shown in Figure 1), followed by training and testing on only the depressurization and repressurization phases.

Full Procedure (Baseline + Depressurization + Repressurization + Recovery)			
Feature Set	Normalization	Performance Metrics ^a^	
		*RMSE (%)*	*R* ^2^
All Features	None	13 ± 2	0.85 ± 0.04
	Baseline	12 ± 3	0.85 ± 0.08
Vital Signs	None	23 ± 3	0.50 ± 0.14
	Baseline	17 ± 2	0.71 ± 0.08
ABP Waveform	None	13 ± 2	0.85 ± 0.04
	Baseline	13 ± 3	0.84 ± 0.08
HRDN only	None	14 ± 3	0.82 ± 0.07
	Baseline	15 ± 5	0.77 ± 0.04
Simulated Hemorrhage (Depressurization)			
Feature Set	Normalization	Performance Metrics ^a^	
		*RMSE (%)*	*R* ^2^
All Features	None	14 ± 2	0.74 ± 0.11
	Baseline	13 ± 4	0.77 ± 0.09
Vital Signs	None	18 ± 3	0.56 ± 0.13
	Baseline	17 ± 2	0.65 ± 0.07
ABP Waveform	None	14 ± 2	0.76 ± 0.10
	Baseline	13 ± 3	0.78 ± 0.09
HRDN only	None	16 ± 3	0.67 ± 0.12
	Baseline	16 ± 3	0.68 ± 0.13
Simulated Resuscitation (Repressurization)			
Feature Set	Normalization	Performance Metrics ^a^	
		*RMSE* (%)	*R* ^2^
All Features	None	14 ± 3	0.74 ± 0.11
	Baseline	16 ± 4	0.67 ± 0.19
Vital Signs	None	21 ± 3	0.44 ± 0.16
	Baseline	15 ± 2	0.71 ± 0.08
ABP Waveform	None	14 ± 3	0.75 ± 0.08
	Baseline	17 ± 6	0.71 ± 0.08
HRDN only	None	13 ± 3	0.80 ± 0.09
	Baseline	18 ± 8	0.56 ± 0.37

^a^. Metrics presented as test set mean ± standard deviation after 5-fold cross validation.

**Table 4:** Receiver Operating Characteristic Area Under Curve (ROC AUC) results for training and testing on the full data set (includes the baseline, depressurization, repressurization, and recovery phases from each experiment as shown in Figure 1) followed by training and testing on just the depressurization and repressurization phases.

Full Procedure (Baseline + Depressurization + Repressurization + Recovery)				
Feature Set	*Normalization*	ROC AUC		
		*CRM ≥ 70%*	*CRM ≥ 40%*	*CRM ≥ 5%*
All Features	None	0.98 ± 0.01	0.98 ± 0.01	0.94 ± 0.01
	Baseline	0.97 ± 0.02	0.96 ± 0.02	0.92 ± 0.03
Vital Signs	None	0.85 ± 0.03	0.93 ± 0.02	0.91 ± 0.02
	Baseline	0.92 ± 0.02	0.96 ± 0.01	0.93 ± 0.02
ABP Waveform	None	0.98 ± 0.01	0.98 ± 0.01	0.95 ± 0.01
	Baseline	0.96 ± 0.04	0.95 ± 0.03	0.91 ± 0.04
HRDN only	None	0.98 ± 0.01	0.97 ± 0.01	0.93 ± 0.02
	Baseline	0.96 ± 0.02	0.94 ± 0.03	0.90 ± 0.04
Simulated Hemorrhage (Depressurization)				
Feature Set	*Normalization*	ROC AUC		
		*CRM ≥ 70%*	*CRM ≥ 40%*	*CRM ≥ 5%*
All Features	None	0.95 ± 0.03	0.95 ± 0.01	0.90 ± 0.04
	Baseline	0.96 ± 0.02	0.94 ± 0.02	0.90 ± 0.03
Vital Signs	None	0.87 ± 0.04	0.92 ± 0.03	0.90 ± 0.04
	Baseline	0.90 ± 0.02	0.93 ± 0.02	0.91 ± 0.03
ABP Waveform	None	0.95 ± 0.03	0.95 ± 0.01	0.93 ± 0.02
	Baseline	0.96 ± 0.02	0.94 ± 0.02	0.89 ± 0.02
HRDN only	None	0.92 ± 0.04	0.92 ± 0.01	0.82 ± 0.08
	Baseline	0.94 ± 0.03	0.91 ± 0.04	0.85 ± 0.08
Simulated Resuscitation (Repressurization)				
Feature Set	Training Scheme	ROC AUC		
	*Normalization*	*CRM ≥ 70%*	*CRM ≥ 40%*	*CRM ≥ 5%*
All Features	None	0.96 ± 0.01	0.96 ± 0.02	0.85 ± 0.04
	Baseline	0.95 ± 0.04	0.95 ± 0.03	0.86 ± 0.03
Vital Signs	None	0.87 ± 0.04	0.88 ± 0.05	0.78 ± 0.06
	Baseline	0.95 ± 0.02	0.95 ± 0.01	0.86 ± 0.02
ABP Waveform	None	0.96 ± 0.02	0.97 ± 0.01	0.86 ± 0.02
	Baseline	0.94 ± 0.05	0.95 ± 0.04	0.85 ± 0.04
HRDN only	None	0.97 ± 0.01	0.97 ± 0.01	0.88 ± 0.03
	Baseline	0.91 ± 0.07	0.90 ± 0.08	0.81 ± 0.06

## Data Availability

The data presented in this study can be made available upon request pending U.S. Department of Defense approval.

## References

[1] Eastridge B.J., Mabry R.L., Seguin P., Cantrell J., Tops T., Uribe P., Mallett O., Zubko T., Oetjen-Gerdes L., Rasmussen T.E. (2012). Death on the Battlefield (2001–2011). J. Trauma Acute Care Surg..

[2] Coppola S., Froio S., Chiumello D. (2014). Fluid Resuscitation in Trauma Patients. Curr. Opin. Crit. Care.

[3] Koons N.J., Nguyen B., Suresh M.R., Hinojosa-Laborde C., Convertino V.A. (2020). Tracking DO_2_ with Compensatory Reserve During Whole Blood Resuscitation in Baboons. Shock.

[4] Huang G.S., Dunham C.M. (2017). Mortality Outcomes in Trauma Patients Undergoing Prehospital Red Blood Cell Transfusion: A Systematic Literature Review. Int. J. Burn. Trauma.

[5] Ravi P.R., Puri B. (2017). Fluid Resuscitation in Haemorrhagic Shock in Combat Casualties. Disaster Mil. Med..

[6] Committee on Tactical Combat Casualty Care (2021). Tactical Combat Casualty Care (TCCC) Guidelines for Medical Personnel.

[7] Mutschler M., Nienaber U., Münzberg M., Wölfl C., Schoechl H., Paffrath T., Bouillon B., Maegele M., Trauma Register D.G.U. (2013). The Shock Index Revisited—A Fast Guide to Transfusion Requirement? A Retrospective Analysis on 21,853 Patients Derived from the TraumaRegister DGU^®^. Crit. Care.

[8] Liu J., Khitrov M.Y., Gates J.D., Odom S.R., Havens J.M., de Moya M.A., Wilkins K., Wedel S.K., Kittell E.O., Reifman J. (2015). Automated Analysis of Vital Signs to Identify Patients With Substantial Bleeding Before Hospital Arrival. Shock.

[9] Hanna K., Harris C., Trust M.D., Bernard A., Brown C., Hamidi M., Joseph B. (2020). Multicenter Validation of the Revised Assessment of Bleeding and Transfusion (RABT) Score for Predicting Massive Transfusion. World J. Surg..

[10] Stewart C.L., Mulligan J., Grudic G.Z., Convertino V.A., Moulton S.L. (2014). Detection of Low-Volume Blood Loss. J. Trauma Acute Care.

[11] Kowalski A., Brandis D. (2022). Shock Resuscitation. StatPearls.

[12] Convertino V.A., Howard J.T., Hinojosa-Laborde C., Cardin S., Batchelder P., Mulligan J., Grudic G.Z., Moulton S.L., MacLeod D.B. (2015). Individual-Specific, Beat-to-Beat Trending of Significant Human Blood Loss. Shock.

[13] Convertino V.A., Koons N.J. (2020). The Compensatory Reserve: Potential for Accurate Individualized Goal-directed Whole Blood Resuscitation. Transfusion.

[14] Li X., Pinsky M.R., Dubrawski A. (2022). Automated Assessment of Cardiovascular Sufficiency Using Non-Invasive Physiological Data. Sensors.

[15] Convertino V.A., Wirt M.D., Glenn J.F., Lein B.C. (2016). The Compensatory Reserve For Early and Accurate Prediction Of Hemodynamic Compromise. Shock.

[16] Hinojosa-Laborde C., Howard J.T., Mulligan J., Grudic G.Z., Convertino V.A. (2016). Comparison of Compensatory Reserve during Lower-Body Negative Pressure and Hemorrhage in Nonhuman Primates. Am. J. Physiol.-Regul. Integr. Comp. Physiol..

[17] Reljin N., Zimmer G., Malyuta Y., Mendelson Y., Darling C.E., Chon K.H. Detection of Blood Loss in Trauma Patients Using Time-Frequency Analysis of Photoplethysmographic Signal. Proceedings of the 2016 IEEE-EMBS International Conference on Biomedical and Health Informatics (BHI).

[18] Techentin R.W., Felton C.L., Schlotman T.E., Gilbert B.K., Joyner M.J., Curry T.B., Convertino V.A., Holmes D.R., Haider C.R. 1D Convolutional Neural Networks for Estimation of Compensatory Reserve from Blood Pressure Waveforms. Proceedings of the 2019 41st Annual International Conference of the IEEE Engineering in Medicine and Biology Society (EMBC).

[19] Kerpel A., Ben-Menachem E., Mandelbaum T., Hofstetter E., Preisman S., Berkenstadt H. (2017). Evaluation of Miniature Dynamic Light Scattering Technology for the Assessment of Hemodynamic Status During Graded Hemorrhage and Retransfusion in Pigs. Mil. Med..

[20] Moulton S.L., Mulligan J., Grudic G.Z., Convertino V.A. (2013). Running on Empty; The Compensatory Reserve Index. J. Trauma Acute Care.

[21] Convertino V.A., Grudic G., Mulligan J., Moulton S. (2013). Estimation of Individual-Specific Progression to Impending Cardiovascular Instability Using Arterial Waveforms. J. Appl. Physiol..

[22] Convertino V.A., Techentin R.W., Poole R.J., Dacy A.C., Carlson A.N., Cardin S., Haider C.R., III D.R.H., Wiggins C.C., Joyner M.J. (2022). AI-Enabled Advanced Development for Assessing Low Circulating Blood Volume for Emergency Medical Care: Comparison of Compensatory Reserve Machine-Learning Algorithms. Sensors.

[23] Koons N.J., Moses C.D., Thompson P., Strandenes G., Convertino V.A. (2022). Identifying Critical DO_2_ with Compensatory Reserve during Simulated Hemorrhage in Humans. Transfusion.

[24] Schiller A.M., Howard J.T., Convertino V.A. (2017). The Physiology of Blood Loss and Shock: New Insights from a Human Laboratory Model of Hemorrhage. Exp. Biol. Med..

[25] Vellido A. (2020). The Importance of Interpretability and Visualization in Machine Learning for Applications in Medicine and Health Care. Neural Comput. Applic..

[26] Gupta J.F., Telfer B.A., Convertino V.A. Feature Importance Analysis for Compensatory Reserve Measurement to Predict Hemorrhagic Shock. Proceedings of the 2022 44th Annual International Conference of the IEEE Engineering in Medicine & Biology Society (EMBC).

[27] Elgendi M. (2012). On the Analysis of Fingertip Photoplethysmogram Signals. Curr. Cardiol. Rev..

[28] Benov A., Yaslowitz O., Hakim T., Amir-Keret R., Nadler R., Brand A., Glassberg E., Yitzhak A., Convertino V.A., Paran H. (2017). The Effect of Blood Transfusion on Compensatory Reserve. J. Trauma Acute Care.

[29] Nadler R., Convertino V.A., Gendler S., Lending G., Lipsky A.M., Cardin S., Lowenthal A., Glassberg E. (2014). The Value of Noninvasive Measurement of the Compensatory Reserve Index in Monitoring and Triage of Patients Experiencing Minimal Blood Loss. Shock.

[30] Imholz B.P.M., Wieling W., Montfrans G.A., van Wesseling K.H. (1998). Fifteen Years Experience with Finger Arterial Pressure Monitoring: Assessment of the Technology. Cardiovasc. Res..

[31] Shaffer F., Ginsberg J.P. (2017). An Overview of Heart Rate Variability Metrics and Norms. Front. Public Health.

[32] Pearson R.K. (2002). Outliers in Process Modeling and Identification. IEEE Trans. Control. Syst. Technol..

[33] Friedman J.H. (2001). Greedy Function Approximation: A Gradient Boosting Machine. Ann. Stat..

[34] Saeb S., Lonini L., Jayaraman A., Mohr D.C., Kording K.P. (2017). The Need to Approximate the Use-Case in Clinical Machine Learning. Gigascience.

[35] Pedregosa F., Varoquaux G., Gramfort A., Michel V., Thirion B., Grisel O., Blondel M., Müller A., Nothman J., Louppe G. (2011). Scikit-Learn: Machine Learning in Python. J. Mach. Learn. Res..

[36] Bland J.M., Altman D.G. (1986). Statistical Methods for Assessing Agreement Between Two Methods of Clinical Measurement. Lancet.

[37] Krouwer J.S. (2008). Why Bland–Altman Plots Should Use X, Not (Y + X)/2 When X Is a Reference Method. Stat. Med..

[38] Rosenberg A.J., Kay V.L., Anderson G.K., Sprick J.D., Rickards C.A. (2021). A Comparison of Protocols for Simulating Hemorrhage in Humans: Step versus Ramp Lower Body Negative Pressure. J. Appl. Physiol..

[39] Hinojosa-Laborde C., Shade R.E., Muniz G.W., Bauer C., Goei K.A., Pidcoke H.F., Chung K.K., Cap A.P., Convertino V.A. (2014). Validation of Lower Body Negative Pressure as an Experimental Model of Hemorrhage. J. Appl. Physiol..

[40] Schauer S.G., April M.D., Arana A.A., Maddry J.K., Escandon M.A., Linscomb C., Rodriguez D., Convertino V.A. (2021). Efficacy of the Compensatory Reserve Measurement in an Emergency Department Trauma Population. Transfusion.

